# Co-MoO_3_ Nanoparticles Supported on Carbon Nanotubes for Highly Efficient Hydrogen Production from Ammonia Borane

**DOI:** 10.3390/ma18204692

**Published:** 2025-10-13

**Authors:** Xingchi Ma, Xigang Du, Hongyu Liu

**Affiliations:** School of Chemistry and Chemical Engineering, Henan University of Science and Technology, Luoyang 471023, China; 18838749198@163.com

**Keywords:** ammonia borane, cobalt, molybdenum trioxide, hydrolysis, carbon nanotubes

## Abstract

Ammonia borane (AB) is recognized as a highly promising material for hydrogen storage owing to its exceptional safety and high hydrogen density, enabling controllable hydrogen release at room temperature through catalytic hydrolysis. The development of efficient catalysts to accelerate this process remains a critical research challenge. In this work, carbon nanotube (CNT)-supported Co-MoO_3_ nanoparticles were synthesized through reduction with sodium borohydride. The catalyst with a Co/MoO_3_ molar ratio of 1.0:0.1 (denoted as Co_1_Mo_0.1_/CNTs) showed optimal performance in AB hydrolysis, with a turnover frequency (TOF) of 19.15 mol_H2_ mol_cat_^−1^ min^−1^ and an activation energy (E_a_) of 26.41 kJ mol^−1^. The superior performance of the Co_1_Mo_0.1_/CNTs catalyst can be ascribed to the efficient proton-transfer promotion by carboxylated carbon nanotubes and the synergistic catalytic effect between Co and Mo in the system. This study offers a viable pathway for constructing high-efficiency noble metal-free catalysts for hydrogen production from AB hydrolysis.

## 1. Introduction

Energy is a fundamental material basis for the survival and advancement of human society. Fossil resources such as coal, oil, and natural gas have driven the evolution of civilization and sustained global economic growth since the Industrial Revolution [1]. However, worsening pollution and shrinking fossil-fuel supplies are making it imperative to embrace cleaner, more sustainable sources of energy. Hydrogen energy, as a promising secondary energy source, has emerged as a highly anticipated solution amidst conventional energy crises and the ongoing development of renewable energy alternatives. The combustion of hydrogen yields only water as a byproduct, making it the cleanest burning fuel compared to conventional alternatives. Nevertheless, creating hydrogen storage systems that are both safe and efficient is imperative for establishing a viable hydrogen-based economy.

Ammonia Borane (NH_3_BH_3_, AB) demonstrates an exceptional hydrogen storage capacity with a mass fraction of 19.6 wt% and volumetric density of 0.145 kg H_2_/L [2]. Owing to its superior hydrogen storage and release properties, AB is considered a highly promising chemical hydrogen storage material. Hydrogen can be released from AB via pyrolysis, alcoholysis, or hydrolysis. Notably, catalytic hydrolysis enables controlled hydrogen release, where 1 mol of AB yields 3 equivalents of H_2_ (NH_3_BH_3_ + 2H_2_O → NH_4_BO_2_ + 3H_2_↑). This method offers advantages such as mild reaction conditions and CO-free byproducts (preventing catalyst poisoning), making it a safe and practical hydrogen generation technology [3]. The efficiency of AB hydrolysis largely depends on the catalyst. Although noble metals such as Rh [4], Pt [5], and Ru [6] exhibit high catalytic activity for complete AB hydrolysis, their scarcity and high cost challenges hinder large-scale industrial applications. In contrast, non-noble metal catalysts such as Co [7,8], Cu [9,10], and Ni [11] have gained attention due to their cost-effectiveness and abundance. Additionally, compared with noble metal catalysts, non-noble systems exhibit lower intrinsic activity and tend to aggregate. To address these limitations, doping with additional elements (oxides [12], phosphides [13], or other metals) to form composite catalysts has been explored. This strategy increases the number of active sites, reduces the reaction’s activation energy, and enhances catalytic performance in AB hydrolysis. The synergistic interaction between multiple metals modifies the catalyst’s particle morphology and electronic properties, exposing more active sites and promoting efficient electronic synergy.

In addition, studies show that supporting catalysts boost activity and stability via catalyst–support interactions. Carbon-based materials (such as carbon nanotubes [14], graphene [15], graphite skeleton porous carbon materials [16], etc.) have been widely used as catalyst supports in AB hydrolysis due to their high specific surface area, tunable pore structure, and chemical stability. Carbon nanotubes (CNTs) are graphene layers with an outer diameter in the range of 1 to 100 nanometers. Depending on the number of graphene layers, they can be categorized as single-walled (SWCNT) or multi-walled (MWCNT) [17]. Their high surface area makes them excellent catalyst supports, facilitating extensive nanoparticle dispersion and enhancing reactant accessibility to active sites. Furthermore, their mesoporous structure improves mass transfer between reactants and active sites, significantly boosting catalytic activity [18].

In this study, we synthesized CNTs-supported Co catalysts doped with MoO_3_ (Co-MoO_3_/CNTs) through a simple co-reduction approach employing NaBH_4_ as the reductant. The prepared Co_1_Mo_0.1_/CNTs catalyst exhibited excellent catalytic performance in AB hydrolysis. Under ambient conditions, it achieved complete hydrogen release within 3 min, with an initial turnover frequency (TOF) value of 19.15 mol_H2_ mol_cat_^−1^ min^−1^ and an activation energy (E_a_) value of 26.41 kJ mol^−1^.

## 2. Experimental

### 2.1. Materials and Catalysts Characterization

Ammonia borane (NH_3_BH_3_, AB, 97%, Aldrich, St. Louis, MO, USA), sodium borohydride (NaBH_4_, 99%, Aldrich, St. Louis, MO, USA), cobalt chloride hexahydrate (CoCl_2_∙6H_2_O, 99%, Tianjin Bo di Chemical Co., Ltd., Tianjin, China), sodium molybdate dihydrate (Na_2_MoO_4_∙2H_2_O, 99%, Tianjin Chemical Reagent Factory No. 4. Tianjin, China) and multi-walled carbon nanotubes (MWCNTs, carboxylation, Shenzhen Sui heng Technology Co., Ltd., Shenzhen, China) were utilized as received. All reactions were carried out using deionized water as the solvent.

The structure of the samples was studied by transmission electron microscopy (TEM, JEM-2100F, JEOL Ltd., Akishima, Japan) with an accelerating voltage of 200 kV, a point resolution 0.23 nm, and line resolution 0.10 nm. For TEM analysis, nanoparticle suspensions were drop-cast (1–2 droplets) onto carbon-supported copper grids. Elemental maps were obtained using an EDAX ELite scanning transmission electron microscope-energy dispersive spectrometer (STEM-EDS, JEM-2100F, JEOL Ltd., Akishima, Japan). X-ray diffraction (XRD) measurements were carried out on a Rigaku RINT-2200 X-ray diffractometer (Akishima, Japan) with a Cu Kα source, operating at 40 kV and 20 mA. X-ray photoelectron spectroscopy (XPS, Thermo Scientific K-Alpha, ThermoFisher, Waltham, MA, USA) measurements were acquired after Ar sputtering for 2 min with an ESCALABMKLL X-ray photoelectron using an Al Kα source. Fourier transform infrared spectroscopy (FT-IR) used the Shimadzu IRTracer-100 infrared spectrometer (Kyoto, Japan); the sample was prepared by potassium bromide compression method.

### 2.2. Synthesis of Catalysts

The Co-MoO_3_/CNTs catalyst was synthesized via a straightforward one-pot co-reduction approach under ambient conditions. For example, 1 mL of 0.1 mol/L CoCl_2_∙6H_2_O, 0.1 mL of 0.1 mol/L Na_2_MoO_4_∙2H_2_O, and 5 mg of CNTs were dispersed in a pre-cleaned round-bottomed flask. The total volume was brought to 5 mL using deionized water. Subsequently, 15 mg of the reducing agent NaBH_4_ was introduced into the mixture under vigorous stirring until gas evolution ceased, indicating complete reduction. The final black precipitate was collected via centrifugation. To prevent oxidation, the newly prepared catalyst was weighed and immediately re-dispersed in 5 mL water for corresponding catalytic experiments. The obtained catalyst, designated as Co_1_Mo_0.1_/CNTs, could be directly employed for AB dehydrogenation without further treatment. For comparison, Co/CNTs, unsupported Co_1_Mo_0.1_ catalysts, and Co-MoO_3_/CNTs catalysts with other ratio variants including Co_1_Mo_0.2_/CNTs and Co_1_Mo_0.3_/CNTs were also prepared following the same procedure.

### 2.3. Catalytic Measurements

Catalytic hydrogen generation experiments were conducted in a 50 mL round-bottom flask containing 5 mL of the as-prepared catalyst suspension, immersed in a temperature-controlled water bath. The system was connected to an inverted gas burette for real-time hydrogen quantification. A concentrated sulfuric acid scrubber was installed between the two units to neutralize ammonia and other volatile gases. Upon addition of a predetermined amount of AB to the reaction vessel under vigorous magnetic stirring at 800 rpm, the reaction commenced immediately. Hydrogen evolution was monitored by measuring water displacement in the gas burette at regular time intervals. The reaction was considered complete when no further gas generation was observed.

## 3. Results and Discussion

### 3.1. Preparation and Characterization

**Figure 1:**
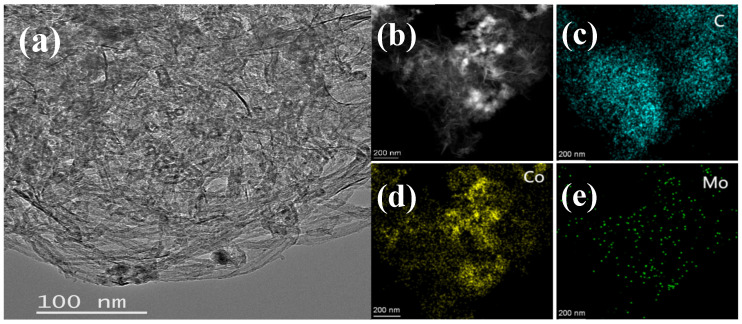
(**a**) TEM images of Co_1_Mo_0.1_/CNTs and (**b**–**e**) STEM-EDS of elemental mapping image, C, Co, and Mo, respectively, for Co_1_Mo_0.1_/CNTs.

**Figure 2:**
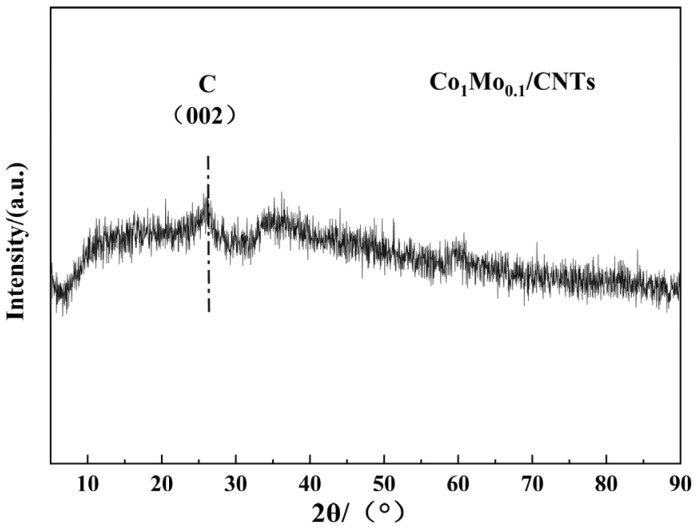
XRD pattern of Co_1_Mo_0.1_/CNTs.

The as-synthesized Co_1_Mo_0.1_/CNTs was systematically characterized by TEM, STEM-EDS, XRD, XPS, and FT-IR techniques. Figure 1a demonstrates that nanoparticles were successfully and uniformly loaded onto the carbon nanotubes. This indicates that the loading process had good consistency. Elemental mapping via STEM-EDS shown in Figure 1c–e indicates that C, Co, and Mo elements achieved relatively stable and uniform dispersion within the material. This significant finding confirms that Co and Mo elements were successfully attached to the carbon nanotubes. Moreover, the EDS analysis revealed a Co/Mo atomic ratio of 91:9 and a Co loading of 0.45 wt% on CNTs, closely matching the theoretical value, thereby confirming the precise synthesis control of Co_1_Mo_0.1_/CNTs. The XRD pattern (Figure 2) exhibits a distinct reflection at 26.43° associated with the crystallographic plane of (002) for carbon, while no discernible diffraction peaks for crystalline Co or Mo phases are observed. This suggests that the metallic components likely exist in non-crystalline or amorphous forms [19]. The absence of sharp metal-related diffraction peaks may be attributed to either the nanoscale dimensions of the metallic species or their non-crystalline nature, both of which typically result in weakened XRD signals.

**Figure 3:**
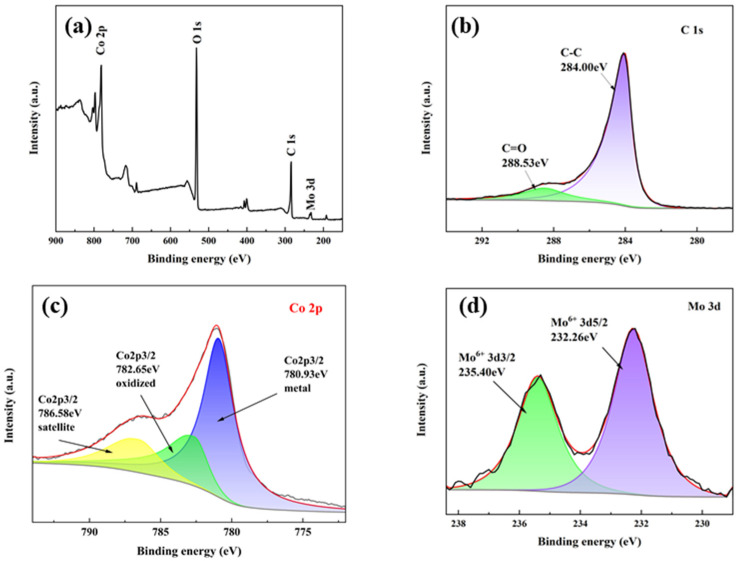
XPS spectra of (**a**) overall spectrum, (**b**) C 1s, (**c**) Co 2p, and (**d**) Mo 3d for as-synthesized Co_1_Mo_0.1_/CNTs. Black lines represent the experimental data obtained from characterization; red lines show the XPS peak-fitting curves.

The chemical states of Co and Mo in the catalyst were thoroughly investigated through XPS analysis (Figure 3). All spectra were calibrated by setting the C 1s peak to 284.00 eV (graphitic carbon). Through careful deconvolution of the high-resolution Co 2p and Mo 3d spectra, we precisely determined the valence states and relative abundances of these elements. Figure 3a presents the XPS overall spectrum, which reveals the types of elements present in the catalyst. The peaks located at 780.93, 531.84, 284.00, and 232.26 eV in the spectrum are attributed to Co 2p, O 1s, C 1s, and Mo 3d, respectively. The C 1s spectrum from Figure 3b displays two contributions at 284.00 and 284.53 eV, assigned to C-C and C=O of C. In the Co 2p [8] region of Figure 3c, the main peak at 780.93 eV is characteristic of the Co 2p_3/2_ metal state and the main peak at 782.65 eV is representative of the Co 2p_3/2_ oxidized state [20]. Additionally, the peak observed at 786.58 eV corresponds to the satellite peak of these principal peaks. After deconvolution of the Co 2p XPS peaks and correction with sensitivity factors, the surface metallic cobalt accounts for 60.72% and Co^2+^ for 39.28%. The Mo 3d spectrum in Figure 3d shows doublet peaks at 232.26 (3d_5/2_) and 235.40 eV (3d_3/2_), indicative of the Mo^6+^ of MoO_3_. The presence of these characteristic peaks provides direct evidence of the chemical state of Co and Mo. By comparison with the standard spectra Co at 778.2 eV and Mo^6+^ 3d at 233.1 eV, the Co 2p binding energy showed a positive shift, while that of Mo 3d was negatively shifted. This phenomenon may stem from the electronic interaction between molybdenum and cobalt, where the high electronegativity of molybdenum may draw more electron density, thereby affecting the electronic environment of cobalt and causing its binding energy to increase. Conversely, the decrease in molybdenum’s binding energy could be due to its lower oxidation state or different chemical environment in the compound compared to cobalt.

**Figure 4:**
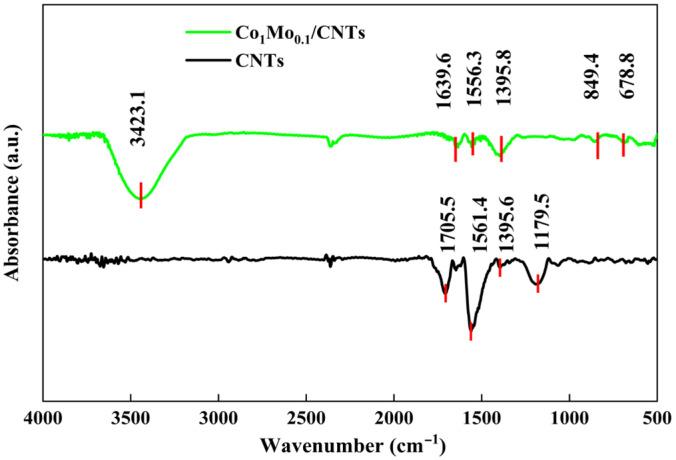
FT-IR of Co_1_Mo_0.1_/CNTs and CNTs.

The FT-IR spectrums of Co_1_Mo_0.1_/CNTs and CNTs are shown in Figure 4. The absorption peak observed at approximately 3423.1 cm^−1^ is associated with O–H stretching of water molecules adsorbed on the surface during the preparation of Co_1_Mo_0.1_/CNTs. The peaks at 678.8 cm^−1^ may arise from Co–O bonds [21], while the peak at about 849.4 cm^−1^ is likely due to Mo–O–Mo vibrations of MoO_3_ in the Co_1_Mo_0.1_/CNTs composite [22]. Peaks around 1705.5 and 1639.6 cm^−1^ correspond to C=C stretching, and those at 1561.4 and 1556.3 cm^−1^ are assigned to C=O stretching [23]. The features at 1395.6, 1179.5, and 1395.8 cm^−1^ are indicative of carboxyl (COOH^-^) groups [24]. The difference in transmittance for C=O and carboxyl between Co_1_Mo_0.1_/CNTs and CNTs may result from the interaction of C=O with H^+^, OH^−^, and free oxygen in water to form carboxyl groups [25].

### 3.2. Catalytic Hydrolysis of AB

**Figure 5:**
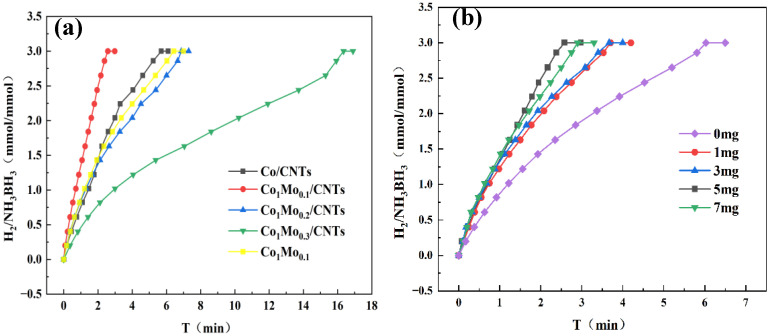
(**a**) Hydrogen generation from AB hydrolysis catalyzed by different catalysts with 31.5 mg AB at 298 K. (**b**) Hydrogen generation from AB hydrolysis catalyzed by different CNTs-supported catalysts with 31.5 mg AB at 298 K.

The hydrolytic dehydrogenation of AB was catalyzed by the synthesized materials and monitored via a water-displacement apparatus. A series of prepared Co_1_Mo_x_/CNTs catalysts with varying Mo contents (x = 0.0, 0.1, 0.2, 0.3) were evaluated at 298 K. As illustrated in Figure 5a, the undoped Co/CNTs finished the hydrolysis of AB within 3 min. Interestingly, upon introducing Mo, the catalytic activity initially improved with increasing Mo content (x = 0.1), but subsequently declined at higher Mo loadings (x > 0.1). This volcano-type trend suggests that excessive Mo may block active Co sites, while an optimal Mo doping level (x = 0.1) enhances the catalytic performance. The carbon nanotube support was found to significantly promote AB hydrolysis. Among all tested catalysts, Co_1_Mo_0.1_/CNTs demonstrated the highest activity, achieving a turnover frequency (TOF) of 19.15 mol_H2_ mol_cat_^−1^ min^−1^. The TOF calculation was based on surface metallic Co on Co_1_Mo_0.1_/CNTs. Comparative data for TOF and E_a_ values across different cobalt-based catalysts are summarized in Table 1. As shown in Table 1, the prepared Co_1_Mo_0.1_/CNTs catalyst exhibited outstanding catalytic activity, even higher than the activity of some precious metal catalysts.

CNTs excel as catalyst supports due to their high conductivity and surface-to-volume ratio, ensuring dense active-site exposure. To optimize the CNT loading amount, systematic experiments were performed with different CNT contents (0, 1, 3, 5, and 7 mg). As evidenced in Figure 5b, catalytic activity shows a positive correlation with CNT content up to 5 mg. Remarkably, the catalyst containing 5 mg CNTs demonstrated optimal performance for AB hydrolysis at 298 K, indicating an ideal dispersion of metal nanoparticles on the CNT support. However, excessive CNT loading (e.g., 7 mg) led to the observed activity decline.

**Figure 6:**
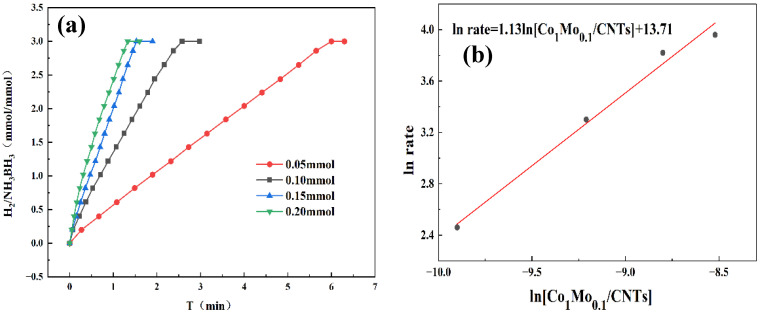
(**a**) Hydrogen production from AB hydrolysis catalyzed by 31.5 mg AB at 298 K under varying Co_1_Mo_0.1_/CNTs catalysts loadings. (**b**) First-order reaction curve of ln rate versus ln[Co_1_Mo_0.1_/CNTs].

**Figure 7:**
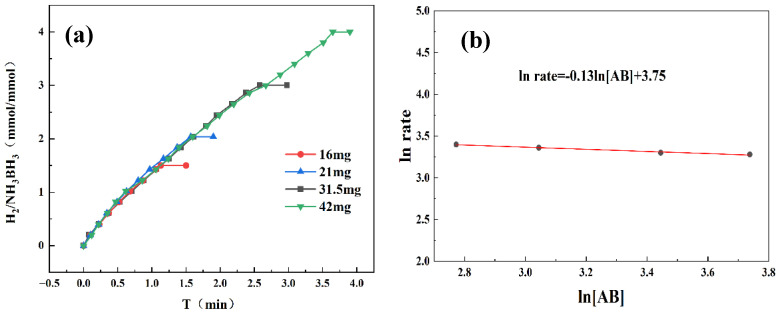
(**a**) Hydrogen production from AB hydrolysis catalyzed by Co_1_Mo_0.1_/CNTs at 298 K under varying AB quality. (**b**) Zero-order reaction curve of ln rate versus ln[AB].

By systematically varying catalyst and AB concentrations, we determined the reaction order and gained mechanistic insight into AB hydrolysis. As demonstrated in Figure 6a, the hydrogen evolution rate exhibited a progressive enhancement with increasing catalyst concentration. Kinetic analysis in Figure 6b reveals a slope of 1.13, confirming first-order dependence on catalyst concentration. Higher catalyst concentrations supplied more active sites, enlarging the catalyst–reactant interface and accelerating the reaction. Therefore, the increase in catalyst concentration has a significant positive impact on enhancing the kinetic behavior of chemical reactions. Figure 7a reveals that hydrogen was released continuously and steadily throughout the reaction, with its generation rate almost unaffected by fluctuations in AB concentration. As shown in Figure 7b, the linear fit yields a slope of 0.13, consistent with zero-order reaction kinetics. This also indicates that the AB hydrolysis reaction was independent of the AB concentration. And the catalyst played a dominant role in the reaction. A possible reason for this is that the active sites on the catalyst surface may already have been fully occupied by AB molecules. Even if the concentration of AB had been further increased, there were no additional active sites available for adsorption; therefore, the reaction rate did not increase accordingly.

**Figure 8:**
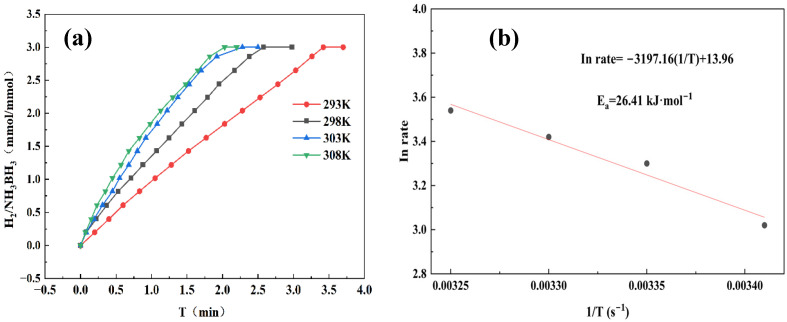
(**a**) Hydrogen production from AB hydrolysis catalyzed by Co_1_Mo_0.1_/CNTs with 31.5 mg AB at different temperatures. (**b**) Corresponding Arrhenius plot of ln rate versus 1/T (s^−1^).

To accurately determine the activation energy (E_a_) for AB hydrolysis, we performed temperature-dependent kinetic studies at 293, 298, 303, and 308 K. As seen in Figure 8a, as the reaction temperature increased step by step, the rate of hydrogen generation also increased. This phenomenon clearly indicates that higher reaction temperatures significantly promote the hydrolysis rate of AB. Through Arrhenius analysis shown in Figure 8b, the Co_1_Mo_0.1_/CNTs catalyst demonstrates a small E_a_ of 26.41 kJ mol^−1^, indicating its high catalytic efficiency for AB hydrolysis.

**Figure 9:**
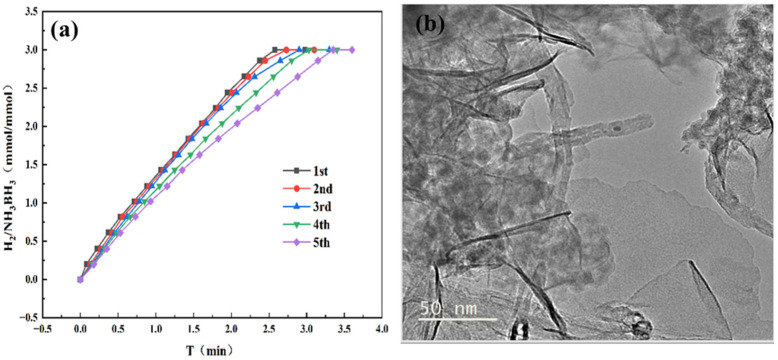
(**a**) Cycle stability test for hydrogen generation via AB hydrolysis catalyzed by Co_1_Mo_0.1_/CNTs adding 31.5 mg AB at 298 K. (**b**) TEM images of Co_1_Mo_0.1_/CNTs catalyst after five cycles.

Catalyst reusability represents a key factor for sustainable hydrogen generation from AB hydrolysis. After the first reaction was completed, we added an additional 31.5 mg of AB to the reaction mixture. This procedure was repeated over five consecutive runs, with the corresponding performance data presented in Figure 9a. Following five consecutive reaction cycles, the catalyst retained 70% of its initial activity, demonstrating the outstanding durability and recyclability of the Co_1_Mo_0.1_/CNTs catalyst. Figure 9b reveals that catalyst detachment from CNTs or particle agglomeration leads to deactivation after five cycles.

**Figure 10:**
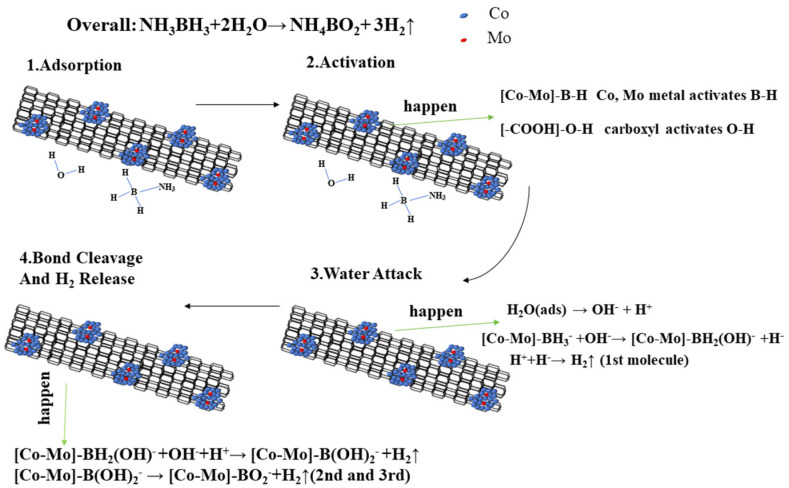
Proposed mechanism for the hydrolysis of AB catalyzed by Co_1_Mo_0.1_/CNTs.

The catalytic hydrolysis of AB follows a well-established four-step mechanism (Figure 10): (1) AB adsorption on metal sites, (2) B-H bond activation, (3) nucleophilic water attack, and (4) B-O bond formation with concomitant H_2_ release [33]. The metal activates the B–H bond of NH_3_BH_3_ while the carboxyl group simultaneously activates water molecules. Our experiments further demonstrate that the Co_1_Mo_0.1_/CNTs catalyst plays a dual role as both a “charge transfer promoter” and a “bifunctional catalytic site”, as shown in Figure 10. The carbonyl oxygen of -COOH bears a partial negative charge. It can polarize the O-H bond of H_2_O, lower the O-H bond dissociation energy barrier of water, promote its heterolytic cleavage to generate a more nucleophilic OH^−^, and accelerate the nucleophilic attack on the boron atom. When carboxylated carbon nanotubes are decorated with Co_1_Mo_0.1_, -COOH and the metal centers form an “acid–metal” dual active site. This synergy reduces the apparent activation energy and promotes rapid hydrolysis.

## 4. Conclusions

In summary, a series of CoMo/CNTs catalysts were facilely synthesized via a one-step co-reduction method using NaBH_4_ as the reducing agent. Among these, the Co_1_Mo_0.1_/CNTs catalyst demonstrated optimal catalytic performance for hydrogen generation from AB hydrolysis, achieving a remarkable TOF of 19.15 mol_H2_ mol_cat_^−1^ min^−1^ at room temperature. This superior catalytic activity originates from two key factors: the carboxyl-rich surface of CNTs facilitates efficient reactant adsorption, and the strong electronic synergy between Co and Mo species optimizes the catalytic active sites. This work provides a cost-effective and highly active catalytic system for generating hydrogen, highlighting the importance of both surface engineering and bimetallic cooperation in catalyst design.

## Figures and Tables

**Table 1:** TOF and E_a_ for hydrogen production via catalytic hydrolysis of AB using different catalysts.

Catalyst	T/K	n_metal/_n(AB)	TOF/mol_H2_ mol_cat_^−1^min^−1^	E_a_/kJ·mol^−1^	Ref.
Co/NPCNW	298	0.075	7.29	25.4	[26]
Co@ N-C-700	298	0.057	5.6	31	[27]
Co/HPC	298	0.11	2.94	32.8	[28]
Co@C-N@SiO2-800	298	/	8.4	36.1	[29]
Cu_3_P-Co_2_P	298	0.1	4.03	33.6	[30]
[Pd(2-pymo)_2_]_n_	298	0.01	1.71	/	[31]
Fe_3_O_4_/SiO_2_−NH_2_/HB@Pd	298	/	2.13	34	[32]
Co_1_Mo_0.1_/CNTs	298	0.1	19.15	26.41	This work

## Data Availability

The original contributions presented in this study are included in the article. Further inquiries can be directed to the corresponding authors.

## References

[1] Jeuland M., Fetter T.R., Li Y., Pattanayak S.K., Usmani F., Bluffstone R.A., Chávez C., Girardeau H., Hassen S., Jagger P. (2021). Is energy the golden thread? A systematic review of the impacts of modern and traditional energy use in low- and middle-income countries. Renew. Sust. Energy Rev..

[2] Liu M.M., Zhou L., Luo X.J., Wan C., Xu L.X. (2020). Recent advances in noble metal catalysts for hydrogen production from ammonia borane. Catalysts.

[3] Sun Q.M., Wang N., Xu Q., Yu J.H. (2020). Nanopore-supported metal nanocatalysts for efficient hydrogen generation from liquid-phase chemical hydrogen storage materials. Adv. Mater..

[4] Shen J., Yang L., Hu K., Luo W., Cheng G. (2015). Rh nanoparticles supported on graphene as efficient catalyst for hydrolytic dehydrogenation of amine boranes for chemical hydrogen storage. Int. J. Hydrogen Energy.

[5] Chen W., Li D., Wang Z., Qian G., Sui Z., Duan X., Zhou X., Yeboah I., Chen D. (2017). Reaction mechanism and kinetics for hydrolytic dehydrogenation of ammonia borane on a Pt/CNT catalyst. AICHE J..

[6] Fan G., Liu Q., Tang D., Li X., Bi J., Gao D. (2016). Nanodiamond supported Ru nanoparticles as an effective catalyst for hydrogen evolution from hydrolysis of ammonia borane. Int. J. Hydrogen Energy.

[7] Zhang J.R., Jia Y.Q., Chu F., Lei N., Bi J.P., Qin H.Y., Liu M.L., Jia Y.X., Zhang L., Jiang L. (2025). Carbothermal shock fabrication of CoO-Cu_2_O nanocomposites on N-doped porous carbon for enhanced hydrolysis of ammonia borane. Rare Metals.

[8] Wu H., Liu L., Liu X., Bian L., Chen Y., Fan Y., Liu B. (2025). In situ construction of Co–Mo2C on N-doped carbon for efficient hydrogen evolution from ammonia borane hydrolysis. Int. J. Hydrogen Energy.

[9] Yuan Y., Chen X., Zhang X., Wang Z., Yu R. (2020). A mof-derived CuCo(O)@ carbon-nitrogen framework as an efficient synergistic catalyst for the hydrolysis of ammonia borane. Inorg. Chem. Front..

[10] Zhang W., Liu J., Wang J., Dong Y., Liu J., Li X. (2025). Construction of heterostructured CuO–Co3O4 catalyst for hydrogen evolution from ammonia borane hydrolysis. J. Phys. Chem. Solids.

[11] Wang C., Tuninetti J., Wang Z., Zhang C., Ciganda R., Salmon L., Moya S., Ruiz J., Astruc D. (2017). Hydrolysis of ammonia-borane over Ni/ZIF-8 nanocatalyst: High efficiency, mechanism, and controlled hydrogen release. J. Am. Chem. Soc..

[12] Ren X., Lv H., Yang S., Wang Y., Li J., Wei R., Xu D., Liu B. (2019). Promoting effect of heterostructured NiO/Ni on Pt nanocatalysts toward catalytic hydrolysis of ammonia borane. J. Phys. Chem. Lett..

[13] Xu C., Yang L., Liu Z., Tao Y. (2024). RuCo@P core-shell nanoparticles filled with carbon nanotubes for highly effective catalytic hydrolysis of ammonia borane. Int. J. Energy Res..

[14] Esteves L.M., Smarzaro J.L., Caytuero A., Oliveira H.A., Passos F.B. (2019). Catalyst preparation methods to reduce contaminants in a high-yield purification process of multiwalled carbon nanotubes. Braz. J. Chem. Eng..

[15] Zou A., Xu X., Zhou L., Lin L., Kang Z. (2021). Preparation of graphene-supported Co-CeOx nanocomposites as a catalyst for the hydrolytic dehydrogenation of ammonia borane. JFCT.

[16] Merve A., Eken K.S., Önder M. (2023). The rational design of gCN/a-WO_x_/Pt heterostructured nanophotocatalysts for boosting the hydrogen generation from the hydrolysis of ammonia borane under visible light. Int. J. Hydrogen Energy.

[17] Singh P., Samorì C., Toma F.M., Bussy C., Nunes A., Al-Jamal K.T., Ménard-Moyon C., Prato M., Kostarelos K., Bianco A. (2011). Polyamine functionalized carbon nanotubes: Synthesis, characterization, cytotoxicity and siRNA binding. J. Mater. Chem..

[18] Li S.F., Guo Y.H., Sun W.W. (2010). Platinum nanoparticle functionalized CNTs as nanoscaffolds and catalysts to enhance the dehydrogenation of ammonia-borane. J. Phys. Chem. C.

[19] Wang Y., Zou K., Zhang D., Li G., Meng W., Wang D., Cao Z., Zhang K., Wu S. (2020). Co–Mo–B nanoparticles supported on carbon cloth as effective catalysts for the hydrolysis of ammonia borane. Int. J. Hydrogen Energy.

[20] Biesinger M.C., Payne B.P., Grosvenor A.P., Lau L.W.M., Gerson A.R., Smart R.S.C. (2011). Resolving surface chemical states in XPS analysis of first row transition metals, oxides and hydroxides: Cr, Mn, Fe, Co and Ni. Appl. Surf. Sci..

[21] Petitto S.C., Marsh E.M., Carson G.A., Langell M.A. (2007). Cobalt oxide surface chemistry: The interaction of CoO(100), Co_3_O_4_ (110) and Co_3_O_4_ (111) with oxygen and water. J. Mol. Catal. A-Chem..

[22] Liang R., Cao H., Qian D. (2011). MoO_3_ nanowires as electrochemical pseudocapacitor materials. Chem. Commun..

[23] Nasser A.G.A.E., Metwally M.G., Shoukry A.A., Nashar R.M.E. (2024). Application of recycled battery graphite decorated with poly hippuric acid/multiwalled carbon nanotubes as an ecofriendly sensor for serotonin. Sci. Rep..

[24] Tomar D., Chaudhary S., Jena K.C. (2019). Self-assembly of l-phenylalanine amino acid: Electrostatic induced hindrance of fibril formation. RSC Adv..

[25] Bychko I., Strizhak P. (2018). Carbon nanotubes catalytic activity in the ethylene hydrogenation. Fuller. Nanotub. Carbon Nanostruct..

[26] Zhou L., Meng J., Li P., Tao Z., Mai L., Chen J. (2017). Ultrasmall cobalt nanoparticles supported on nitrogen-doped porous carbon nanowires for hydrogen evolution from ammonia borane. Mater. Horizons.

[27] Wang H., Zhao Y., Cheng F., Tao Z., Chen J. (2016). Cobalt nanoparticles embedded in porous N-doped carbon as long-life catalysts for hydrolysis of ammonia borane. Catal. Sci. Technol..

[28] Zhang X.L., Zhang D.X., Chang G.G., Ma X.C., Wu J., Wang Y., Yu H.Z., Tian G., Chen J., Yang X.Y. (2019). Bimetallic (Zn/Co) mofs-derived highly dispersed metallic Co/HPC for completely hydrolytic dehydrogenation of ammonia–borane. Ind. Eng. Chem. Res..

[29] Chen M., Xiong R., Cui X., Wang Q., Liu X. (2019). SiO_2_-encompassed Co@N-doped porous carbon assemblies as recyclable catalysts for efficient hydrolysis of ammonia borane. Langmuir.

[30] Li L., Hu H., Zhang L., Qiu J., Feng Y., Liao J. (2022). Cu_3_P-Co_2_P nanoplatelet catalyst towards ammonia borane hydrolysis for hydrogen evolution. Catal. Lett..

[31] Augustyniak A.W., Trzeciak A.M. (2022). Hydrogen production and transfer hydrogenation of phenylacetylene with ammonia borane in water catalyzed by the [Pd(2-pymo)2]n framework. Inorganica Chim. Acta.

[32] Melek T. (2023). Magnetically isolable Pd(0) nanoparticles supported on surface functionalized Fe3O4 for hydrogen generation via ammonia borane hydrolysis. ChemistrySelect.

[33] Liu Q., Ran W., Bao W., Li Y. (2025). A review on catalytic hydrolysis of ammonia borane for hydrogen production. Energies.

